# Point‐of‐care testing can achieve same‐day diagnosis for infants and rapid ART initiation: results from government programmes across six African countries

**DOI:** 10.1002/jia2.25677

**Published:** 2021-03-21

**Authors:** Caroline E Boeke, Jessica Joseph, Melody Wang, Zelalem M Abate, Charles Atem, Khady Diatou Coulibaly, Adisu Kebede, Brianán Kiernan, Leonard Kingwara, Phibeon Mangwendeza, Tatenda Maparo, Rose Nadege Mbaye, Solomon Mukungunugwa, Catherine Ngugi, Divine Nzuobontane, Marie Claire Okomo Assoumou, Yemsirach Reta, Barbara Wambugu, Maria R Rioja, Trevor Peter, Naoko Doi, Lara Vojnov, Shaukat Khan, Jilian A Sacks

**Affiliations:** ^1^ Clinton Health Access Initiative Boston MA USA; ^2^ Clinton Health Access Initiative Addis Ababa Ethiopia; ^3^ Clinton Health Access Initiative Yaoundé Cameroon; ^4^ Division de la Lutte Contre le SIDA et les IST Ministère de la Santé et de l’Action Sociale Dakar Senegal; ^5^ Ethiopian Public Health Institute Addis Ababa Ethiopia; ^6^ Clinton Health Access Initiative Dakar Senegal; ^7^ National HIV Reference Lab Nairobi Kenya; ^8^ National AIDS and STI Control Programme (NASCOP) Nairobi Kenya; ^9^ Clinton Health Access Initiative Harare Zimbabwe; ^10^ Ministry of Health and Child Care Harare Zimbabwe; ^11^ National Public Health Laboratory Yaoundé Cameroon

**Keywords:** point of care, early infant diagnosis, Africa, ART initiation, turnaround times

## Abstract

**Introduction:**

Point‐of‐care (POC) early infant diagnosis (EID) testing has been shown to dramatically decrease turnaround times from sample collection to caregiver result receipt and time to ART initiation for HIV‐positive infants compared to centralized laboratory testing. As governments in sub‐Saharan Africa implement POC EID technologies, we report on the feasibility and effectiveness of POC EID testing and the impact of same‐day result delivery on rapid ART initiation within national programmes across six countries.

**Methods:**

This pre‐/post‐evaluation compared centralized laboratory‐based (pre) with POC (post) EID testing in 52 facilities across Cameroon, Democratic Republic of Congo, Ethiopia, Kenya, Senegal and Zimbabwe between April 2017 and October 2019 (country‐dependent). Data were collected retrospectively from routine records at health facilities for all infants tested under two years of age. Hazard ratios and 95% confidence intervals were calculated to compare time‐to‐event outcomes, visualized with Kaplan–Meier curves, and the Somers’ D test was used to compare continuous outcomes.

**Results:**

Data were collected for 2892 EID tests conducted on centralized laboratory‐based platforms and 4610 EID tests on POC devices with 127 (4%) and 192 (4%) HIV‐positive infants identified, respectively. POC EID significantly reduced the time from sample collection to caregiver result receipt (POC median: 0 days, IQR: 0 to 0 vs. centralized: 35 days, IQR: 26 to 56) and time from sample collection to ART initiation for HIV‐positive infants (POC median: 1 day, IQR: 0 to 7 vs. centralized: 39 days, IQR: 26 to 57). With POC testing, 72% of infants received results on the same day as sample collection; HIV‐positive infants with a same‐day diagnosis had six times the rate of ART initiation compared to those diagnosed one or more days after sample collection (HR: 6.39; 95% CI: 3.44 to 11.85).

**Conclusions:**

Same‐day diagnosis and treatment initiation for infants is possible with POC EID within routine government‐led and ‐supported public sector healthcare facilities in resource‐limited settings. Given that POC EID allows for rapid ART initiation, aligning to the World Health Organization’s recommendation of ART initiation within seven days, its use in public sector programmes has the potential to reduce overall mortality for infants with HIV through early treatment initiation.

## 1 INTRODUCTION

Gaps remain in coverage of early infant diagnosis (EID) of HIV (estimated at 60% globally in 2019) [1], and delays in result delivery can lead to loss‐to‐follow‐up or delayed ART initiation, resulting in increased mortality in HIV‐positive infants [2]. Point‐of‐care (POC) testing for EID has been shown to dramatically improve the timeliness of determining HIV status and reduce HIV‐related morbidity and mortality in infants. POC EID testing was shown initially to be feasible in public sector facilities in Mozambique [3] and several partner supported studies across different country contexts have demonstrated POC testing leads to faster receipt of test results by caregivers and faster ART initiation [4, 5, 6, 7, 8, 9]. As such, the 2016 WHO Consolidated Guidelines on the use of antiretroviral drugs for treating and preventing HIV infection recommends immediate implementation of POC EID. However, the real‐world impact of POC EID on same‐day diagnosis and rapid treatment initiation within decentralized national programmes in sub‐Saharan Africa is still unclear.

Across 52 public sector facilities in six countries, a pre‐/post‐evaluation was conducted to assess differences in clinic receipt of test results, caregiver receipt of test results and test turnaround times comparing POC EID to centralized EID testing. Secondary analysis included differences in ART initiation and turnaround times. Within the POC arm, an additional secondary analysis was conducted to compare time to ART initiation for infants when caregivers received results on the same day as sample collection versus during subsequent clinic visits, one or more days later. This study is the first of its kind to evaluate large‐scale implementation of POC EID in government‐supported public health facilities.

## 2 METHODS

### 2.1 Study design

In this evaluation of routine programmatic implementation of POC EID, quality of EID service delivery and clinical outcomes were compared for centralized laboratory‐based EID testing and POC EID testing at 52 public sector health facilities across six countries: Cameroon, Democratic Republic of Congo (DRC), Ethiopia, Kenya, Senegal and Zimbabwe. In 33 sites, Xpert^®^ HIV‐1 Qual assays were provided for diagnosis of HIV infection on existing Cepheid GeneXpert devices (Cepheid, Sunnyvale, CA, USA) . The remaining 19 sites all used m‐PIMA HIV‐1/2 Detect assays on Abbott m‐PIMA devices (Abbott, Chicago, IL, USA). In Cameroon and Senegal, all the m‐PIMA devices were newly placed at the facility; in Zimbabwe, four devices were newly placed, four sites had existing m‐PIMA devices and three sites had additional new m‐PIMA devices placed in other wards apart from the HIV clinic, that is alternative entry points. The study aimed to compare a six‐month time period in the same facilities before and after POC implementation, although due to logistical constraints in some countries the actual timeframes varied from three to nine months, with data collected between April 2017‐October 2019 (country‐dependent, see Table 1). For all countries except Ethiopia, the pre‐implementation period was exactly one year prior to POC implementation. Sites in DRC and several sites in Senegal did not have centralized testing data available for comparison due to sporadic or poor access to the centralized laboratory and limited documentation of EID tests, but were included in POC‐only analyses. No facilities received support from implementing partners for POC EID.

**Table 1:** Characteristics of the nine studies in six countries implementing POC EID testing

	Cameroon‐1	Cameroon‐2	DRC	Ethiopia	Kenya	Senegal‐1	Senegal‐2	Zimbabwe‐1	Zimbabwe‐2
Number of facilities	3	4	4	9	4	4	4	9	11
Study design	Pre/post	Pre/post	POC only	Pre/post	Pre/post	POC only	Pre/post	Pre/post	Pre/post
Site selection	All facilities running EID tests on GX	All facilities	All facilities running EID tests on GX	All facilities running EID tests on GX	First 4 sites that rolled out GX	All facilities running EID tests on GX	All facilities	All facilities running EID tests on GX	All facilities with pre and post data available
POC device	GX	m‐PIMA	GX	GX	GX	GX	m‐PIMA	GX	m‐PIMA
Device placement in facilities	Existing devices	Placement of new devices	Existing devices	Existing devices	Existing devices	Existing devices	Placement of new devices	Existing devices	Mix of new and existing devices
Time Frame:									
Pre: Centralized Lab	Jul to Dec 2017	Apr to Sep 2017	N/A	Apr to Sep 2018	Oct 2017 to Jun 2018	N/A	Jul to Oct 2018	Oct 2017 to Mar 2018	Jul to Dec 2017
Post: POC	Jul to Dec 2018	Apr to Sep 2018	Jun to Dec 2018	Nov 2018 to Jun 2019	Oct 2018 to Jun 2019	May to Jul 2019	Jul to Oct 2019	Oct 2018 to Mar 2019	Jul to Dec 2018
# of months per time segment	6	6	7	6 (pre) 8 (post)	9	3	4	6	6

DRC, Democratic Republic of Congo; EID, early infant diagnosis; GX: GeneXpert; POC, point‐of‐care.

### 2.2 Patient and site selection

National guidelines dictated eligibility for EID testing, which typically followed the 2015 WHO HIV Testing Guidelines [10]. These guidelines state that infants should be tested at four to six weeks and nine months, or at any point when exhibiting signs or symptoms suggestive of HIV infection. Although global guidance recommends virological testing up to aged 18 months, several national guidelines stipulate that infants can be tested up to 24 months of age due to prolonged breast‐feeding periods. The study cohort included infants who were known to be exposed to HIV, and excluded infants greater than 24 months of age, already known HIV‐positive, or too sick to be tested. If infants happened to be tested twice during either time period, only their first valid test record was included.

POC device placement for initial implementation in each country was determined by Ministries of Health based on infrastructure, human resource availability, existing POC device placement and utilization rates and annual patient volumes. For this evaluation, all implementing facilities were included except in Kenya, where only the first four sites were included in the evaluation.

### 2.3 Implementation of POC EID testing

Implementation included training health facility staff on operational procedures for testing, clinical utilization of test results, documentation, device maintenance and quality control by national reference laboratory staff. Cartridges were supplied for POC tests. Supportive supervision visits were conducted throughout the implementation period to monitor activities, ensure enough staff were trained and troubleshoot any challenges with POC devices. The existing staff conducted all activities associated with POC testing.

GeneXpert devices in health facilities across all countries were already being used for TB testing in the onsite laboratory and were operated by laboratory staff, whereas m‐PIMA devices were placed in the onsite laboratory in Cameroon and Senegal. In Zimbabwe, m‐PIMA devices were either placed in the clinic, in antenatal, maternity, paediatric and/or outpatient wards, or in the on‐site laboratory. As a result, Zimbabwe’s facilities had a wider variety of health cadres operating POC devices, including nurses and trained diagnostic assistants in the wards. Testing was done according to specific device manufacturers’ instructions. All facilities used whole blood as a sample type for GeneXpert and m‐PIMA. In laboratories where GeneXpert was used for multiple test types, for example EID and TB, prioritization of tests was at the discretion of laboratory staff; in most facilities, EID samples were prioritized due to this test being the most time sensitive.

### 2.4 Data collection and analysis procedures

Trained data collectors retrospectively extracted demographic, service delivery and clinical data from national and facility‐specific laboratory and clinic registers as well as patient charts; data were captured electronically via SurveyCTO (2016 Dobility, Inc., Cambridge, MA, USA). Data collection for both centralized and POC EID outcomes allowed for each infant to have a follow‐up period of 90 days, to allow for complete documentation in facility registers of testing and, for those who tested HIV‐positive, treatment outcomes. In Senegal, due to logistical constraints, 20 infants tested on m‐PIMA only had 30 days of follow‐up, not the full 90 days.

The World Health Organization (WHO) recommends that results are returned to the caregiver as soon as possible, no more than 28 days after sample collection [11]. Therefore, the primary outcome was the proportion of test results received by caregiver within 28 days of sample collection. Caregiver receipt of results within the same day, seven days and ninety days was also analysed. The WHO also recommends rapid ART initiation, defined as ART initiation within seven days of diagnosis, for HIV‐positive children under five years [12]. Thus, the proportion of infants initiating ART within the same day and seven, twenty‐eight and ninety days of sample collection was also analysed for HIV‐positive infants, as were turnaround times between steps in the cascade of care. Finally, among infants tested with POC devices, median time from sample collection to ART initiation was compared between infants receiving a positive diagnosis on the same day as sample collection versus at least one day or more after sample collection.

Statistical analysis was performed using Stata SE 15 (StataCorp, TX, USA) and limited to infants with a valid test result (invalid or missing test results were excluded), as well as to those facilities with both POC and centralized testing data. Patients with missing dates for steps in the cascade of care were considered as failing to complete that step during analysis. Testing characteristics were presented as numbers and proportions for categorical variables and median and interquartile range (IQR) for continuous variables. The Somers’ D test was used to compare continuous outcomes (i.e. turnaround times) for centralized versus POC testing, to account for facility‐level clustering. Time‐to‐event analyses were conducted with comparisons between centralized and POC testing, visually displayed in Kaplan–Meier curves, and through calculation of hazard ratios and 95% confidence intervals using maximum likelihood estimation and parametric regression survival‐time models, as the proportional hazards assumption was not met. Shared frailties [13] were utilized to account for facility‐level clustering and country was adjusted for as a covariate in the model. Statistical significance was defined as *p* < 0.05.

### 2.5 Ethical approvals

Approval for this study was obtained from a local Institutional Review Board in each country (Cameroon: Comite National D’Ethique de la Recherche Pour La Sante Humaine 2018/06/1041/CE/CNERSH/SP; Democratic Republic of Congo: Université de Kinshasa Ecole de Sante Publique Comite D’Ethique ESP/CE/079/2019; Ethiopia: Ethiopian Public Health Institute: EPHI‐IRB‐060‐2017; Kenya: Kenyatta National Hospital‐University of Nairobi Ethics & Research Committee P197/03/2018; Senegal: Republique du Senegal Ministere de la Sante et de L’Action Sociale 269MSAS/DGS/DLM/DLSI; Zimbabwe: Medical Research Council of Zimbabwe MRCZ/A/2470) and Advarra Institutional Review Board in the United States (#Pro000252217). Written informed consent was waived as data were collected retrospectively and all clinical elements (i.e. EID testing) followed standard of care practice.

## 3 RESULTS

Patient and facility characteristics are described in Table 2 (disaggregated by country in Table S1). Overall, 4610 POC EID and 2892 centralized laboratory‐based EID tests were conducted of which 97% (4479) and 92% (2653) had valid test results respectively. Error rates were similar, with 2% of POC test results and 1% of centralized results being errors or invalid, but there were significantly fewer missing results for POC compared to centralized tests (1% vs. 7%). HIV positivity rate was 4% (192) for POC tests and 4% (127) for centralized tests. Overall, the median age of infants at sample collection was 45 (IQR: 35 to 64) days during the POC implementation and 46 (IQR: 42 to 58) days during centralized testing. 50% of POC tests were among females as were 41% of centralized tests. Meanwhile, the median age of HIV‐positive infants at sample collection was 149 days (IQR: 52 to 270) for infants tested on POC compared to 157 days (IQR: 64 to 267) with centralized testing.

**Table 2:** Characteristics of infants receiving EID testing

	Centralized		POC	
	n	%/median (IQR)	n	%/median (IQR)
Total tests	2892	100%	4610	100%
Country/study				
Cameroon‐1	160	6%	197	4%
Cameroon‐2	191	6%	315	7%
DRC	NA	NA	49	1%
Ethiopia	143	5%	136	3%
Kenya	186	6%	76	2%
Senegal‐1	NA	NA	18	0%
Senegal‐2	12	0%	53	1%
Zimbabwe‐1	488	17%	668	14%
Zimbabwe‐2	1712	59%	3098	67%
Infant age (days)	2862	46 (42 to 58)	4532	45 (35 to 64)
Infant age, category				
0 to 2 months	2186	76%	3333	72%
3 to 8 months	489	17%	847	18%
9 to 18 months	179	6%	329	7%
19 to 24 months	8	0%	23	0%
Missing	30	1%	78	2%
Infant sex				
Female	1200	41%	2327	50%
Male	1332	46%	2206	48%
Missing	360	12%	77	2%
Test results				
Negative	2226	87%	4287	93%
Positive	127	4%	192	4%
Invalid/error	30	1%	94	2%
Missing	209	7%	37	1%
Entry point				
PMTCT/MCH	2481	86%	4072	88%
Other	271	9%	500	11%
Missing	140	5%	38	1%
Device				
GeneXpert	NA	NA	1144	25%
m‐PIMA	NA	NA	3466	75%

EID, early infant diagnosis; IQR, interquartile range; NA, not applicable; PMTCT/MCH, prevention of mother‐to‐child transmission/maternal child health; POC, point‐of‐care.

**Figure 1:**
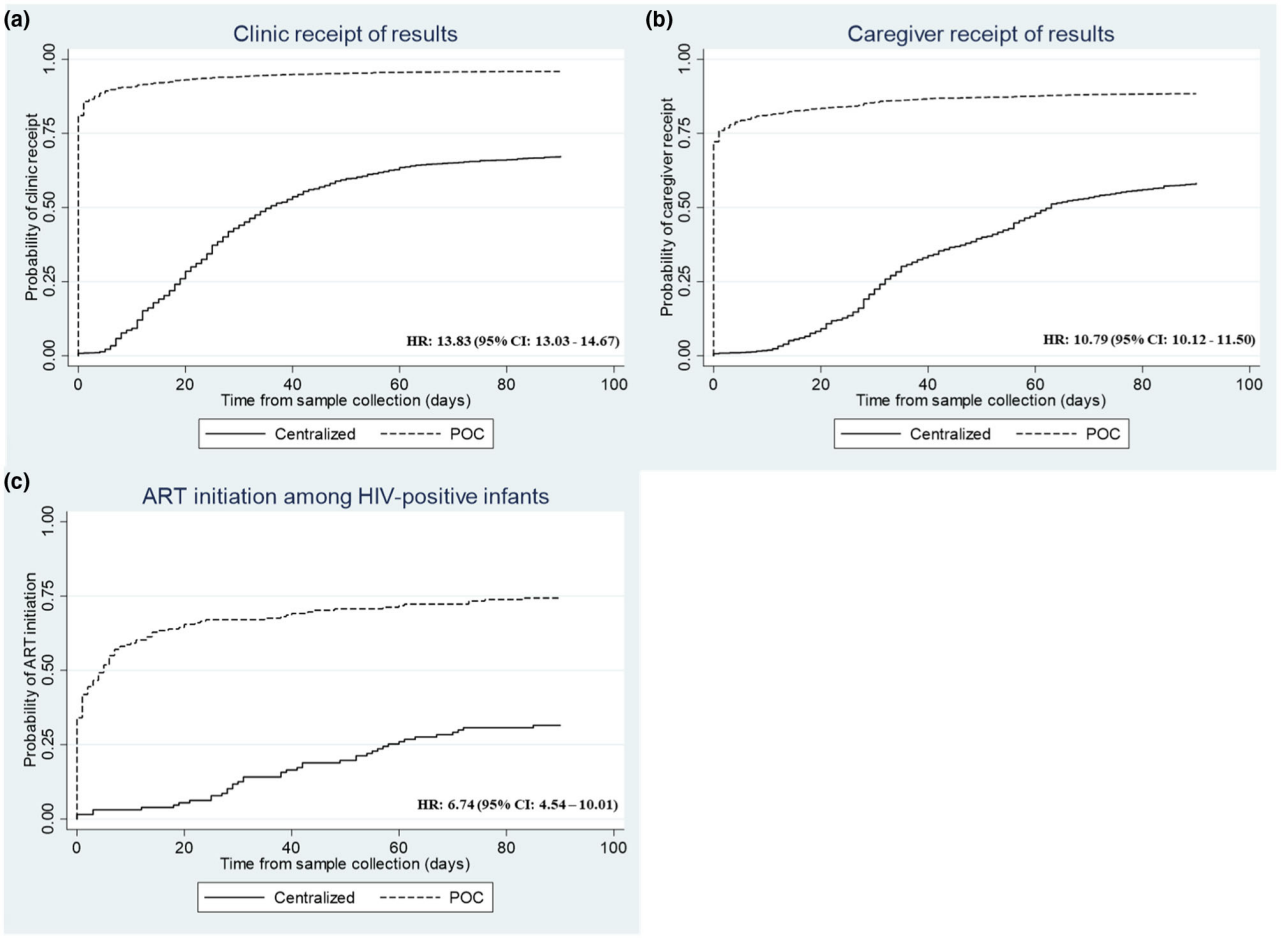
Kaplan–Meier curves and hazard ratios (95% confidence intervals) for time from sample collection to: **(a)** clinic receipt of results comparing POC EID testing and centralized EID testing; **(b)** caregiver receipt of results comparing POC EID testing and centralized EID testing; and **(c)** ART initiation comparing POC EID testing and centralized EID testing

The median turnaround time from sample collection to clinic receipt of result was same day (IQR: 0 to 0) with POC testing in comparison to 24 days (IQR: 14 to 36) with centralized testing. The median turnaround time to caregiver receipt also decreased to same day (IQR: 0 to 0) with POC testing from 35 days (IQR: 26 to 56) with centralized testing (Table 3). There was a near‐fourteen times increase in time to results return to clinic (HR: 13.83; 95% CI: 13.03 to 14.67) and near‐eleven times increase in time to results return to caregiver (HR: 10.79; 95% CI: 10.12 to 11.50) with POC compared to centralized testing (Table 3; Figure 1a and b). Seventy‐two percent of POC EID results were received by the caregiver on the same day of sample collection, and the proportion increased to 80% at seven days, 85% at 28 days and 88% at 90 days. With centralized testing, 58% of caregivers received results within 90 days. For the median turnaround times from sample collection to clinic and caregiver receipt of results, and time to ART initiation, there were some differences between countries using POC EID, most notably in Kenya, which had a median turnaround time to caregiver receipt of results of 28 days (IQR: 14 to 32) (Table S2). In general, while there was some heterogeneity in country contexts and in outcomes across countries, an improvement with POC EID was seen consistently across countries.

**Table 3:** Comparison^a^ of centralized laboratory and point‐of‐care early infant diagnosis on turnaround times^b^ and proportions from sample collection to clinic and caregiver receipt of results and ART initiation, with hazard ratios and 95% confidence intervals for time‐to‐event analyses

	Centralized		POC		*p*‐value	HR (95% CI)
	N	n (%)/median (IQR)	N	n (%)/median (IQR)		
Sample collection to clinic receipt						
Turnaround time (days)	1783	24 (14 to 36)	4233	0 (0 to 0)	<0.001	13.83 (13.03 to 14.67)
Proportion result received same‐day	2653	22 (1%)	4413	3570 (81%)	<0.001	
Proportion result received within 7 days	2653	155 (6%)	4413	3982 (90%)	<0.001	
Proportion result received within 28 days	2653	1111 (42%)	4413	4142 (94%)	<0.001	
Proportion result received within 90 days	2653	1783 (67%)	4413	4229 (96%)	<0.001	
Sample collection to caregiver receipt						
Turnaround time (days)	1543	35 (26 to 56)	3902	0 (0 to 0)	<0.001	10.79 (10.12 to 11.50)
Proportion result received same‐day	2653	19 (1%)	4413	3185 (72%)	<0.001	
Proportion result received within 7 days	2653	35 (1%)	4413	3547 (80%)	<0.001	
Proportion result received within 28 days	2653	507 (19%)	4413	3754 (85%)	<0.001	
Proportion result received within 90 days	2653	1543 (58%)	4413	3902 (88%)	<0.001	
Sample collection to ART initiation, HIV + patients						
Turnaround time (days)	40	39 (26 to 57)	142	1 (0 to 7)	<0.001	6.74 (4.54 to 10.01)
Proportion initiated on ART same‐day	127	2 (2%)	191	65 (34%)	0.021	
Proportion initiated on ART within 7 days	127	4 (3%)	191	109 (57%)	<0.001	
Proportion initiated on ART within 28 days	127	13 (10%)	191	128 (67%)	<0.001	
Proportion initiated on ART within 90 days	127	40 (31%)	191	142 (74%)	<0.001	

ART, antiretroviral therapy; CI, confidence interval; HR, hazard ratio; IQR, interquartile range; POC, point‐of‐care.

^a^ Facilities without centralized testing data from the pre‐period were excluded from this analysis, as were test results that were errors, invalid or missing

^b^ Continuous turnaround time results are limited to those who received their results.

The median time from sample collection to ART Initiation was also significantly faster for POC (1 day; IQR: 0 to 7) compared to centralized testing (39 days; IQR: 26 to 57; Table 3). Overall, HIV‐positive infants who received a POC test result had an ART initiation rate more than six times faster than those who were diagnosed through centralized testing (HR: 6.74; 95% CI: 4.54 to 10.01; Table 3; Figure 1c). Within seven days after sample collection, 57% of infants who had tested positive with POC were initiated on treatment compared to only 3% with centralized testing. The proportion of infants initiated within 90 days after sample collection increased to 74% with POC versus 31% with centralized testing.

**Figure 2:**
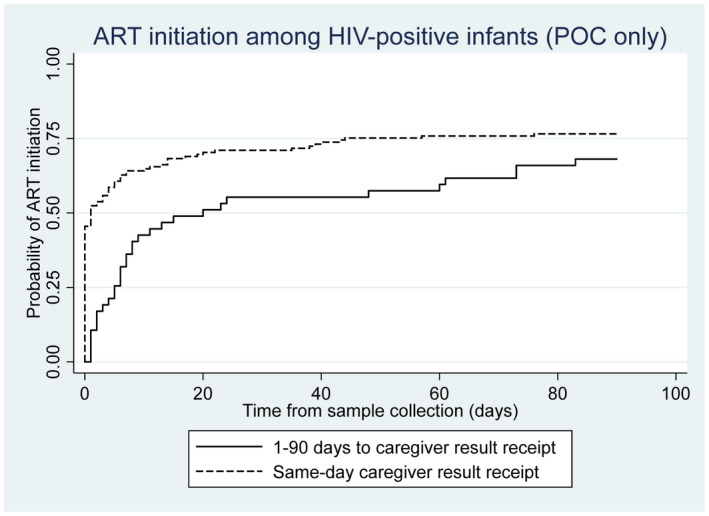
ART initiation among infants testing HIV‐positive on POC platforms only, comparing caregivers receiving test result on the same‐day as sample collection to caregiver result receipt on a subsequent day

Outcomes were analysed separately for infants who received same‐day diagnosis versus infants who received their diagnosis on a subsequent day with POC. Forty‐six percent of HIV‐positive infants who received same‐day diagnosis (caregiver receipt) initiated on ART on the day of sample collection. By seven days, 64% of same‐day diagnosis infants initiated compared to 36% of infants whose caregivers received test results during a subsequent visit. Overall, infants with a same‐day diagnosis as sample collection had an ART initiation rate more than six times faster than those who received their diagnosis on a subsequent day (HR: 6.39; 95% CI: 3.44 to 11.85; Figure 2).

## 4 DISCUSSION

In this assessment of programme implementation across 52 public sector health facilities in six African countries, the use of POC EID testing significantly improved timeliness of clinic and caregiver receipt of results and subsequent ART initiation. Infants who received POC testing had an ART initiation rate more than six times faster than those who were diagnosed through centralized testing. In addition, in analyses limited to HIV‐positive infants tested on POC, receiving results on the same day as sample collection expedited rapid ART initiation (within seven days) compared to infants whose caregivers received the diagnosis on subsequent days. This study adds important implementation knowledge and generalizable results as it was conducted within the existing public health system across six countries with mature and nascent centralized laboratory systems.

These findings demonstrate a dramatic improvement in timely results delivery and rapid initiation of treatment for this vulnerable infant population in public health systems, consistent with previous studies showing benefits of POC EID [4, 6, 7, 8, 9]. Notably, a study in eight African countries from 2014 to 2017 reported that POC EID significantly reduced the median time to caregiver receipt of results from 55 to 0 days and the median time to ART initiation from 49 to 0 days [6]. The sites in this study were supported by the Elizabeth Glaser Pediatric AIDS Foundation (EGPAF) and thus results may not be generalizable to public‐sector facilities; also, this study did not specifically look at the impact of same‐day diagnosis on ART initiation rates. A cluster‐randomized trial in Mozambique reported that POC EID significantly increased ART initiation within 60 days (89.7% vs. 12.8% for centralized testing) and retention in care after 90 days (61.6% vs. 42.9%); the median time from sample collection to ART initiation was less than one day for the POC EID arm [4]. In a stepped‐wedge trial in Kenya and Zimbabwe, POC EID significantly reduced time to caregiver receipt of results and ART initiation [7]. Finally, small feasibility studies in Malawi and Zimbabwe also reported turnaround times to ART initiation of one day or less with POC EID [8, 9]. It is reassuring that in our large study across multiple public‐sector contexts, similarly dramatic improvements were observed, including median turnaround times of 0 days for caregiver receipt of results and 1 day for ART initiation.

Our study demonstrates the positive impact of same‐day result delivery on rapid ART initiation. In contrast to infants, children over two years of age and adults (tested using lateral flow, rapid diagnostic tests) receive HIV diagnosis on the same visit as when testing is performed, which then allows for same‐day treatment initiation. Ideally, this should also be the case for infants who are at a higher risk of morbidity and mortality and would benefit from rapid ART initiation, translating to better health outcomes [14, 15, 16]. Instead, HIV diagnosis for infants was observed to take closer to 28 days with centralized laboratory testing. It is also important to consider the time and cost savings to the caregiver from being able to receive results and initiate an infant on ART in a single day versus having to make multiple trips to the facility for each of these steps. In our study, 46% of infants with same‐day diagnosis initiated on ART the same day thus completing the cascade of care in one visit, and 64% of infants initiated within seven days, as recommended by the WHO. However, gaps in care remain. Only 74% of infants tested through POC ever initiated treatment, underscoring that POC testing may only be one part of the solution. This represents a missed opportunity to improve outcomes in positive infants and suggests that additional interventions outside of implementation of POC testing are needed to support EID programmes to ensure timely initiation of HIV‐positive infants.

While we observed consistent improvements with POC EID across countries, some differences were observed in data from particular countries. First, Zimbabwe had larger testing volumes than other contexts due to a larger number of facilities in the country, the addition of birth testing to EID programmes, and some testing in alternate entry points. The primary difference we observed in POC outcomes was in Kenya, where the median turnaround time from sample collection to caregiver receipt of results (28 days) was substantially higher than in other countries, although this still represented a nearly two‐week decrease compared to testing in the centralized laboratory. The median turnaround time from sample collection to clinic receipt of results was five days in Kenya; this indicates that most results were available at the facility within a week, yet caregivers did not return to collect them for several weeks. These values were higher than those reported in the stepped‐wedge trial of POC EID in Kenya supported by EGPAF [7]. Unlike partner supported sites, there was not an active mechanism for result delivery to caregivers in the context of the public sector sites included in our analysis in Kenya, attenuating the impact of POC. These findings highlight the importance of appropriate programme messaging, clinical mentorship and active follow‐up with patients to maximize the benefits of POC EID, which was noted previously when POC testing was piloted for at/near‐birth testing in Kenya [17].

Additional considerations for governments considering POC EID testing programmes include feasibility and usability of the device, cost and cost‐effectiveness. We observed relatively low error rates in this study (2% for POC, compared to 1% for centralized testing), which is reassuring in a real‐world context. A qualitative study supported by EGPAF reported high levels of satisfaction with POC EID by healthcare workers and laboratory technicians, indicating that POC programmes would be feasible for the national scale [18]. While we do not present information on cost or cost‐effectiveness in this analysis, a recent analysis in Zimbabwe concluded that POC EID testing is likely to be cost‐effective [19] and that benefits will be greater by investing in POC EID compared to strengthening centralized laboratory networks [20, 21]. Additionally, a time‐motion study in Zimbabwe noted that POC did not require additional human resource time versus sending DBS samples for EID testing at a centralized laboratory [22]. Based on mounting evidence in support of POC EID, governments across sub‐Saharan Africa are scaling up POC EID more broadly, with the Ministry of Health of Malawi adopting a policy of providing POC EID testing for all infants.

This evaluation has several limitations, most notably the fact that the study design was not a randomized controlled trial; utilizing a pre/post design meant that other factors, especially those that change over time, could have influenced the outcomes. Testing volumes could not be directly compared for POC versus centralized testing, as some sites had additional testing conducted in the implementation period. A clinical refresher training occurred as part of POC implementation, which did not happen before the lab‐based timeline and could have impacted results. Additionally, while every attempt was made to ensure that the data included was of high quality, as data were captured through routine care, some facilities had large amounts of missing data points, often due to a lack of documentation and poor record keeping. Varying sample sizes in testing volumes across the countries meant that some countries influenced the results more than others (e.g. Zimbabwe contributed 79% of all testing data) and country‐specific analyses could not be conducted in all settings, especially those with very small numbers. Another limitation was that we assessed data 90 days after sample collection, but ART initiation may have occurred after this timeframe in some cases, so we did not capture every case of ART initiation for those in this dataset. We also did not capture visit dates beyond ART initiation, and therefore could not examine retention in care. Finally, in the analysis comparing same‐day diagnosis and diagnosis on a subsequent visit, immortal time bias may be present.

## 5 CONCLUSIONS

These findings support the implementation of POC EID in routine government‐supported public sector healthcare facilities in resource‐limited settings to provide same‐day test results and treatment initiation for infants. Given the large number of infants tested across 52 facilities in six countries, findings may be generalizable to other similar programmes at facilities in sub‐Saharan Africa. While there may be some implementation challenges that need to be addressed and areas for continued improvement and support, POC EID has been demonstrated to dramatically reduce time to results delivery to infant caregivers and infant initiation on treatment, which will likely translate to improvements in infant survival. Most importantly, data shows same‐day diagnosis is achievable in a public health context and leads to a faster rate of ART initiation thus supporting the broader use of POC testing for EID.

## COMPETING INTERESTS

The authors declare no competing interests.

## AUTHORS’ CONTRIBUTIONS

JAS, LV, TP and CEB conceived of and planned the study. MW, SK, JJ, MRR, ZMA, CA, BK and TM led study implementation and data collection. JJ and SK led the data analysis. CEB, JJ and SK wrote the initial manuscript draft. All authors have read and approved the final manuscript.

## Supporting information


**Table S1.** Characteristics of infants receiving EID testing by country
**Table S2.** Comparison of centralized laboratory and point‐of‐care early infant diagnosis on turnaround times^†^ from sample collection to clinic and caregiver receipt and ART initiation, by country
