# Supplementary material for: Point‐of‐care testing can achieve same‐day diagnosis for infants and rapid ART initiation: results from government programmes across six African countries
Source: J Int AIDS Soc. 2021 Mar 21;24(3):e25677. doi: 10.1002/jia2.25677 (PMC7981587; doi:10.1002/jia2.25677)
Supplement: Supplementary file 1 — Table S1. Characteristics of infants receiving EID testing by country Table S2. Comparison of centralized laboratory and point‐of‐care early infant diagnosis on turnaround times† from sample collection to clinic and caregiver receipt and ART initiation, by country [file JIA2-24-e25677-s001.docx]

| **Supplemental Table 1: Characteristics of infants receiving EID testing by country** | | | | |
| --- | --- | --- | --- | --- |
|  | **Centralized** | | **POC** | |
|  | **N** | **n(%) / median (IQR)** | **N** | **n(%) / median (IQR)** |
| **Cameroon** |  |  |  |  |
| Total tests | **351** |  | **512** |  |
| Infant age (days) | 349 | 52 (44 - 94) | 504 | 72 (46 - 211) |
| Infant sex, female | 257 | 112 (44%) | 511 | 249 (49%) |
| Valid results* | 351 | 318 (91%) | 512 | 510 (100%) |
| Positive results | 318 | 12 (4%) | 510 | 27 (5%) |
| Entry point - PMTCT/MCH | 246 | 231 (94%) | 511 | 447 (87%) |
| Device: GX | -- | -- | 512 | 197 (38%) |
| **DRC** |  |  |  |  |
| Total tests | -- | -- | **49** |  |
| Infant age (days) | -- | -- | 47 | 48 (8 - 189) |
| Infant sex, female | -- | -- | 49 | 21 (43%) |
| Valid results | -- | -- | 49 | 49 (100%) |
| Positive results | -- | -- | 49 | 1 (2%) |
| Entry point - PMTCT/MCH | -- | -- | 49 | 46 (94%) |
| Device: GX | -- | -- | 49 | 49 (100%) |
| **Ethiopia** |  |  |  |  |
| Total tests | **143** |  | **136** |  |
| Infant age (days) | 141 | 46 (45 - 64) | 135 | 48 (45 - 76) |
| Infant sex, female | 143 | 79 (55%) | 135 | 72 (53%) |
| Valid results | 143 | 117 (82%) | 136 | 131 (96%) |
| Positive results | 117 | 3 (3%) | 131 | 3 (2%) |
| Entry point - PMTCT/MCH | 143 | 142 (99%) | 136 | 134 (99%) |
| Device: GX | -- | -- | 136 | 136 (100%) |
| **Kenya** |  |  |  |  |
| Total tests | **186** |  | **76** |  |
| Infant age (days) | 183 | 45 (42 - 56) | 75 | 47 (43 - 69) |
| Infant sex, female | 186 | 76 (41%) | 76 | 34 (45%) |
| Valid results | 186 | 176 (95%) | 76 | 74 (97%) |
| Positive results | 176 | 3 (2%) | 74 | 1 (1%) |
| Entry point - PMTCT/MCH | 186 | 183 (98%) | 72 | 72 (100%) |
| Device: GX | -- | -- | 76 | 76 (100%) |
| **Senegal** |  |  |  |  |
| Total tests | **12** |  | **71** |  |
| Infant age (days) | 12 | 7 (58%) | 71 | 145 (45 - 203) |
| Infant sex, female | 12 | 12 (100%) | 71 | 34 (48%) |
| Valid results | 12 | 1 (8%) | 71 | 70 (99%) |
| Positive results | 12 | 12 (100%) | 70 | 2 (3%) |
| Entry point - PMTCT/MCH | 12 | 7 (58%) | 67 | 67 (100%) |
| Device: GX | -- | -- | 71 | 18 (25%) |
| **Zimbabwe** |  |  |  |  |
| Total tests | **2200** |  | **3766** |  |
| Infant age (days) | 2177 | 45 (42 - 54) | 3700 | 44 (7 - 52) |
| Infant sex, female | 1934 | 926 (48%) | 3691 | 1917 (52%) |
| Valid results | 2200 | 2030 (92%) | 3766 | 3645 (97%) |
| Positive results | 2030 | 108 (5%) | 3645 | 158 (4%) |
| Entry point - PMTCT/MCH | 2165 | 1913 (88%) | 3737 | 3306 (88%) |
| Device: GX | -- | -- | 3766 | 668 (18%) |
| *Valid results exclude test results that were errors, invalid, or missing | | | |  |
| EID: early infant diagnosis; POC: point-of-care; IQR: interquartile range; PMTCT/MCH: prevention of mother-to-child transmission/maternal child health; GX: GeneXpert | | | | |
|  |  |  |  |  |

| **Supplemental Table 2: Comparison of centralized laboratory and point-of-care early infant diagnosis on turnaround times**† **from sample collection to clinic and caregiver receipt and ART initiation, by country** | | | | | | |
| --- | --- | --- | --- | --- | --- | --- |
|  |  |  |  |  |  |  |
|  | **Centralized** | | **POC** | |  |  |
|  | **n** | **median (IQR)** | **n** | **median (IQR)** | **p-value** |  |
| Time from sample collection to clinic receipt of results | | | |  |  |  |
| Cameroon | 83 | 40 (14-57) | 433 | 0 (0-1) | 0.202 |  |
| DRC | -- | -- | 48 | 2 (1-4) | -- |  |
| Ethiopia | 85 | 48 (40-61) | 131 | 0 (0-0) | <0.001 |  |
| Kenya | 159 | 25 (16-34) | 70 | 5 (3-17) | 0.026 |  |
| Senegal | 3 | 83 (82-89) | 32 | 1 (0-2) | <0.001 |  |
| Zimbabwe | 1453 | 22 (13-34) | 3567 | 0 (0-0) | <0.001 |  |
| **Overall*** | **1783** | **24 (14-36)** | **4233** | **0 (0-0)** | **<0.001** |  |
| Time from sample collection to caregiver receipt of results | | | |  |  |  |
| Cameroon | 163 | 44 (27-62) | 431 | 0 (0-3) | <0.001 |  |
| DRC | -- | -- | 49 | 2 (1-5) | -- |  |
| Ethiopia | 55 | 56 (48-70) | 128 | 0 (0-0) | <0.001 |  |
| Kenya | 102 | 42 (29-58) | 61 | 28 (14-32) | 0.006 |  |
| Senegal | 0 | -- | 23 | 1 (0-2) | -- |  |
| Zimbabwe | 1223 | 33 (25-53) | 3259 | 0 (0-0) | <0.001 |  |
| **Overall*** | **1543** | **35 (26-56)** | **3902** | **0 (0-0)** | **<0.001** |  |
| Time from sample collection to ART initiation | | |  |  |  |  |
| Cameroon | 5 | 52 (28-56) | 24 | 0 (0-5) | 0.146 |  |
| DRC | -- | -- | 1 | 0 (0-0) | -- |  |
| Ethiopia | 2 | 50 (42-58) | 1 | 1 (1-1) | -- |  |
| Kenya | 3 | 29 (18-61) | 0 | -- | -- |  |
| Senegal | 0 | -- | 2 | 38 (2-73) | -- |  |
| Zimbabwe | 30 | 38 (25-55) | 115 | 1 (0-7) | <0.001 |  |
| **Overall*** | **40** | **38.5 (26-56.5)** | **142** | **1 (0-7)** | **<0.001** |  |
| *Overall excludes DRC and Senegal's GeneXpert sites from POC as they have no centralized test comparator  †Continuous turnaround time results are limited to those who received their results | | | | | |  |
| POC: point-of-care; CI: confidence interval; IQR: interquartile range; ART: antiretroviral therapy | | | | | |  |
